# Retinal Nerve Fiber Layer Thickness in Women with Polycystic Ovary Syndrome

**DOI:** 10.1155/2013/752186

**Published:** 2013-11-26

**Authors:** Mehmet Demir, Dilek Guven, Arzu Koc, Savas Ozdemir, Efe Can

**Affiliations:** ^1^Department of Ophthalmology, Sisli Etfal Training and Research Hospital, Sisli, 34400 Istanbul, Turkey; ^2^Department of Obstetrics and Gynecology, Sisli Etfal Training and Research Hospital, Sisli, 34400 Istanbul, Turkey

## Abstract

*Aim*. To compare the retinal nerve fiber layer (RNFL) thickness between women with polycystic ovary syndrome (PCOS) and healthy women. *Materials and Methods*. The study included 88 eyes of 44 women (group 1) with PCOS and 84 eyes of 42 healthy women (group 2). In all subjects, the RNFL and ganglion cell complex (GCC) thicknesses were measured by optical coherence tomography (OCT). In addition, visual acuity (VA), intraocular pressure (IOP), refractive errors, central macular thickness (CMT), central corneal thickness (CCT), and excavation of optic disc were evaluated in all subjects. *Results*. Mean values of GCC, IOP, VA, CMT, CCT, and refractive errors were similar between the 2 groups. The average RNFL, superior average RNFL, and inferior average RNFL thicknesses were higher in subjects with PCOS than in healthy subjects (*P* = 0.003, *P* = 0.012, and *P* = 0.009), respectively. *Conclusion*. The average RNFL, superior average RNFL, and inferior average RNFL thicknesses in women with PCOS were significantly higher than in healthy women.

## 1. Introduction

Polycystic ovary syndrome (PCOS) is one of the most common endocrinopathies among women in the reproductive age, and it affects 5–7% of this group [1]. It is characterized by an irregular menstrual cycle, ovulatory dysfunction, and hyperandrogenism [2, 3]. Metabolic alterations, insulin resistance, and obesity are often in the patient with PCOS [4]. More than 40% of women with PCOS might develop impaired glucose tolerance or type 2 diabetes mellitus [5, 6]. Optical coherence tomography (OCT) is a noninvasive, noncontact technique that utilizes near infrared, low coherence light passing through a Michelson interferometer to obtain two-dimensional images of the retina, the resolution of which is approximately 5–10 *μ*m in the axial plane [7].

## 2. Materials and Methods

This study included 88 eyes of 44 women (group 1) with PCOS and 84 eyes of 42 healthy women (group 2). The women with PCOS were recruited from among those who visited the Department of Obstetrics and Gynecology. The women with PCOS were selected according to the Androgen Excess and PCOS Society (AES 2006) criteria that included hyperandrogenism, ovulatory dysfunction, and/or polycystic ovary morphology [8]. The women who had a systemic problem other than PCOS were not included in the study. The control group included healthy women who had no ophthalmologic and systemic disorders. Informed consent was obtained from all included subjects, and the study protocol was approved by the local Ethical Committee. Further, all procedures were performed in accordance with the Declaration of Helsinki. The anterior-posterior segments, visual acuity (VA), intraocular pressure (IOP), central macular thickness (CMT), central corneal thickness (CCT), and cup/disc ratio (c/d) were examined for all patients in the ophthalmology clinic (Table 1). In addition, superior nasal (SN) RNFL, nasal upper (NU) RNFL, nasal lateral (NL) RNFL, inferior nasal (IN) RNFL, inferior temporal (IT) RNFL, temporal lateral (TL) RNFL, temporal upper (TU) RNFL, superior temporal (ST) RNFL, average (Avg.) RNFL, superior (Sup.) Avg. RNFL, inferior (Inf.) Avg. RNFL, Avg. ganglion cell complex (GCC), Sup. GCC and Inf. GCC were measured (Table 2).

Exclusion criteria for both groups included presence of glaucoma, ocular hypertension (IOP > 21 mmHg), diabetes mellitus, retinal vasculitis or dystrophy, and history of intraocular surgery, amblyopia, or refractive errors more than ±1.00 diopter spherical equivalent. RNFL, CMT, CCT, and GCC thicknesses were measured using OCT (RTVue-100; Optovue, Fremont, CA). CCT was measured by ultrasonic pachymeter in all patients. Independent *t*-test and Mann-Whitney *U* test were used for statistical evaluation. A *P* value of <0.05 was considered statistically significant.

## 3. Results

The mean age (mean ± SD) of the PCOS group was 24.55 ± 5.67 (range: 18–34) years and that of the control group was 27.29 ± 5.9 (range: 18–33) years (*P* = 0.173).

Mean VA, IOP, CMT, CCT, and C/D were similar in both groups (Table 1). The RNFL average was 109.98 ± 8.19 *μ*m in the study group and 103.96 ± 8.28 in the control group (*P* = 0.003). The average superior RNFL thickness was 111.55 ± 10.36 *μ*m in the study group and 105.39 ± 9.11 *μ*m in the control group (*P* = 0.012); the average inferior RNFL thickness was 108.32 ± 8.49 *μ*m in the study group and 102.64 ± 9.08 *μ*m in the control group (*P* = 0.009). Also mean SN RNFL, IN RNFL, and ST RNFL were thicker in women with PCOS than in healthy women (Table 2).

## 4. Discussion

We aimed to compare the measurements of RNFL, IOP, CCT, and GCC in women with PCOS and healthy women.

In this study, which is the first of its kind, we have analyzed the thicknesses of RNFL, GCC, CCT, CMT, and the IOP in women with PCOS and compared it with healthy women. It is known that in patients with PCOS, the eye also gets affected. Typically, the tear function, drainage and osmolarity [9, 10], meibomian gland function [11], and ocular surface [12] were affected by PCOS. OCT has been used for estimation of RNFL and GCC thicknesses [13–18]. High nerve fiber density was found in PCOS patients [18]. High level of nerve growth factor (NGF) was reported in women with PCOS [19]. Androgens and nerve growth factor have trophic actions on nerves [20–22]. Insulin resistance that can cause diabetic eye changes in women with PCOS. RNFL thickness was thicker in women with PCOS than in healthy women in superior nasal, inferior nasal and superior temporal quadrants. Also average RNFL thickness and average superior and average inferior quadrants RNFL thicknesses were significantly thicker in women with PCOS than in healthy women. The limitations of this study were small sample size, lack of pathologic examination, and inability to reason the results. Studies with larger numbers of participants are required to clarify the clinical and pathophysiological significance of RNFL thickness in women with PCOS. The results of this study can be taken into account in evaluating patients who had PCOS and glaucoma or multiple sclerosis.

In summary, we found that the average RNFL, average superior and inferior RNFL, SN RNFL, IN RNFL, and ST RNFL thicknesses were significantly higher in women with PCOS than in healthy women. We have interpreted that excess androgens and NGF cause an increase in RNFL thickness in women with PCOS.

## Figures and Tables

**Table 1:** Values of age, VA, IOP, CMT, and CCT of study and control groups.

	Study group (44 patients; 88 eyes)	Control group (42 patients; 84 eyes)	*P* ^†^
Age (mean ± SD)	24.55 ± 5.67	27.29 ± 5.9	0.173
VA (logMAR)	0.00	0.00	>0.999
IOP (mm Hg)	16.13 ± 2.34	15.57 ± 3.33	0.423
CMT (*μ*m)	218.5 ± 32.13	228.04 ± 19.56	0.165
CCT (*μ*m)	556.75 ± 30.29	551.88 ± 35.91	0.556
C/D	0.09 ± 0.16	0.09 ± 0.14	0.673*

^†^Independent *t*-test, *Mann-Whitney *U* test, VA: visual acuity, logMAR: logarithm of the minimum angle of resolution, IOP: intraocular pressure, CMT: central macular thickness, CCT: central corneal thickness, and C/D: cup/disc ratio.

**Table 2:** RNFL (*μ*m) and GCC (*μ*m) analysis in both groups.

	Study group (88 eyes)	Control group (84 eyes)	*P* ^†^
SN RNFL	126.18 ± 21.34	115.39 ± 18.85	0.032
NU RNFL	84.93 ± 18.13	81.14 ± 17	0.379
NL RNFL	73.8 ± 13.06	71.36 ± 15.15	0.471
IN RNFL	130.25 ± 23.32	118.25 ± 21.19	0.031
IT RNFL	150.43 ± 14.78	145.39 ± 14.26	0.157
TL RNFL	79.14 ± 16.14	75.57 ± 10.45	0.303
TU RNFL	88.16 ± 13.37	88.54 ± 12.62	0.906
ST RNFL	145 ± 16.07	135.96 ± 15.73	0.022
Avg. RNLF	109.98 ± 8.19	103.96 ± 8.28	0.003
Sup. Avg. RNFL	111.55 ± 10.36	105.39 ± 9.11	0.012
Inf. Avg. RNFL	108.32 ± 8.49	102.64 ± 9.08	0.009
Avg. GCC	98.09 ± 7.33	95.68 ± 5.11	0.133
Sup. GCC	97.34 ± 7.68	95.36 ± 5.3	0.236
Inf. GCC	98.84 ± 7.33	96.11 ± 5.16	0.09

^†^Independent *t*-test, RNFL: retinal nerve fiber layer, GCC: ganglion cell complex, SN: superior nasal, UN: nasal upper, NL: nasal lateral, IN: inferior nasal, IT: inferior temporal, TL: temporal lateral, TU: temporal upper, ST: superior temporal, Avg: average, Sup: superior, Inf: inferior, RNFL: retinal nerve fiber layer, GCC: ganglion cell complex, and UP: upper.
